# Supplementary material for: Extension of a de novo TIM barrel with a rationally designed secondary structure element
Source: Protein Sci. 2021 Mar 20;30(5):982–9. doi: 10.1002/pro.4064 (PMC8040861; doi:10.1002/pro.4064)
Supplement: Supplementary file 1 — Figure S1 Biochemical characterization of all four variants in comparison Table S1: Amino acid sequences of sTIM11noCys and the computationally derived variants Table S2: Motif filters Table S3: Rosetta AbinitioRelax flags Table S4: Rosetta relax flags Table S5: Rosetta enzyme_design flags and corresponding resfile [file PRO-30-982-s001.pdf]

Supplementary Material for:

Extension of a *de novo* TIM barrel with a rationally designed secondary structure element

Jonas Gregor Wiese, Sooruban Shanmugaratnam, Birte Höcker

Content

Figure S1: Biochemical characterization of all four variants in comparison 1
Table S1: Amino acid sequences of sTIM11noCys and the computationally derived variants 2
Table S2: Motif filters 3
Table S3: Rosetta AbinitioRelax flags 4
Table S4: Rosetta relax flags 4
Table S5: Rosetta enzyme_design flags and corresponding resfile 5

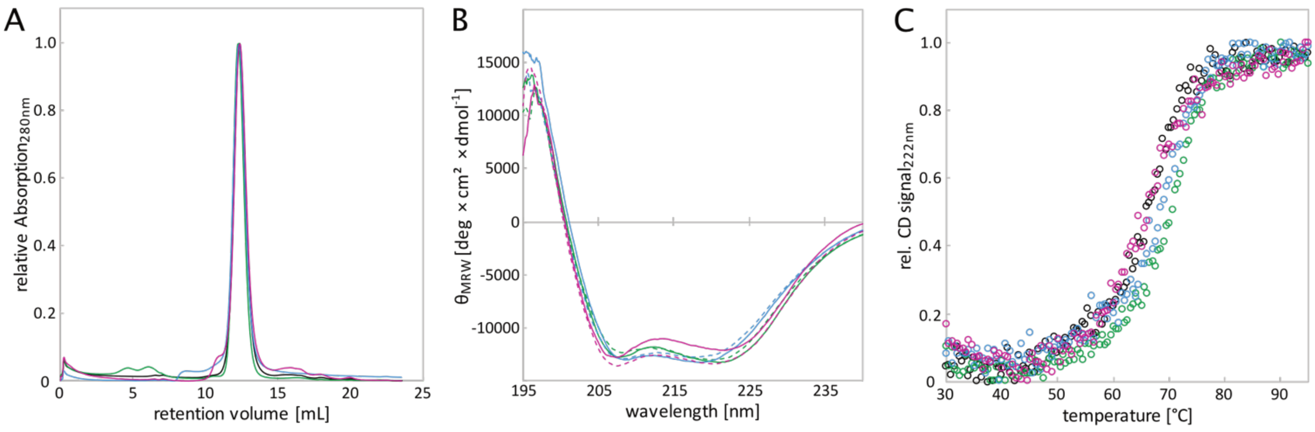

Supplementary Figure 1: Biochemical characterization of all four variants in comparison. (A) Analytical size-exclusion chromatography. (B) Far-UV CD before (solid line) and after thermal denaturation (dashed line). (C) Thermal melting measurements. In all three panels sTIM11_helix1 is shown in green, sTIM11_helix2 in blue, sTIM11_helix3 in black, and sTIM11_helix4 in magenta.

Supplementary Table 1: Amino acid sequences of sTIM11noCys and the computationally derived variants. sTIM11 was modified by removing the symmetry breaking cysteines in the sequence (red) that did not form the expected stabilizing disulfide bond in the original work (Huang et al., 2016). Extensions are highlighted in yellow. The third variant yielded an X-ray structure and is presented in this work

sTIM11noCys	MDKDEAWKQVEQLRREGATQIAYRSDDWRDLKEAWKKGADILIVDAT<br>DKDEAWKQVEQLRREGATQIAYRSDDWRDLKEAWKKGADILIVDAT<br>DKDEAWKQVEQLRREGATQIAYRSDDWRDLKEAWKKGADILIVDAT<br>DKDEAWKQVEQLRREGATQIAYRSDDWRDLKEAWKKGADILIVDAT<br>GLEHHHHHH
sTIM11<br>_helix 1	MDKDEAWKQVEQLRREGATQIAYRSDDWRDLKEAWKKGADILIVDAT<br>DKDEAWKQVEQLRREGATQIAYRSDDWRDLKEAWKKGADILIVDQAEMMQNGMS<br>KDEAWKQVEQLRREGATQIAYRSDDWRDLKEAWKKGADILIVDAT<br>DKDEAWKQVEQLRREGATQIAYRSDDWRDLKEAWKKGADILIVDAT<br>GLEHHHHHH
sTIM11<br>_helix 2	MDKDEAWKQVEQLRREGATQIAYRSDDWRDLKEAWKKGADILIVDAT<br>DKDEAWKQVEQLRREGATQIAYRSDDWRDLKEAWKKGADILIVDEAQMRQNNMP<br>KDEAWKQVEQLRREGATQIAYRSDDWRDLKEAWKKGADILIVDAT<br>DKDEAWKQVEQLRREGATQIAYRSDDWRDLKEAWKKGADILIVDAT<br>GLEHHHHHH
sTIM11<br>_helix 3	MDKDEAWKQVEQLRREGATQIAYRSDDWRDLKEAWKKGADILIVDAT<br>DKDEAWKQVEQLRREGATQIAYRSDDWRDLKEAWKKGADILIVSEEMARHAP<br>KDEAWKQVEQLRREGATQIAYRSDDWRDLKEAWKKGADILIVDAT<br>DKDEAWKQVEQLRREGATQIAYRSDDWRDLKEAWKKGADILIVDAT<br>GLEHHHHHH
sTIM11<br>_helix 4	MDKDEAWKQVEQLRREGATQIAYRSDDWRDLKEAWKKGADILIVDAT<br>DKDEAWKQVEQLRREGATQIAYRSDDWRDLKEAWKKGADILIVGDAKQCRQKGL<br>KDEAWKQVEQLRREGATQIAYRSDDWRDLKEAWKKGADILIVDAT<br>DKDEAWKQVEQLRREGATQIAYRSDDWRDLKEAWKKGADILIVDAT<br>GLEHHHHHH

Supplementary Table 2: Motif filters. After the secondary structure prediction by PSIPRED of the βαβ-fragment containing the randomly generated inserts, the predictions were filtered by regular expressions to obtain α-helical inserts. The leading four dots are placeholders for the first β-strand of the fragment

Target Insert	Regular Expression
4 residue α-helix	....CCHHHHCC*
	....CCCHHHHCC*
	....CCHHHHCCC*
	....CCCHHHHCCC*
5 residue α-helix	....CCHHHHHCC*
	....CCCHHHHHCC*
	....CCHHHHHCCC*
	....CCCHHHHHCCC*
6 residue α-helix	....CCHHHHHHCC*
	....CCCHHHHHHCC*
	....CCHHHHHHCCC*
	....CCCHHHHHHCCC*
7 residue α-helix	....CCHHHHHHHCC*
	....CCCHHHHHHHCC*
	....CCHHHHHHHCCC*
	....CCCHHHHHHHCCC*
8 residue α-helix	....CCHHHHHHHHCC*
	....CCCHHHHHHHHCC*
	....CCHHHHHHHHCCC*
	....CCCHHHHHHHHCCC*

Supplementary Table 3: Rosetta AbinitioRelax flags

AbinitioRelax flags
-database $DATABASE_PATH<br>-in:file:fasta design.fasta<br>-in:file:frag3 $INPUT_FILE_3.3mers<br>-in:file:frag9 $INPUT_FILE_9.9mers<br>-out:file:silent $OUTPUT_FILE.out<br>-nstruct 1000<br>-abinitio:relax

Supplementary Table 4: Rosetta relax flags

relax flags
-in:file:s $PDB_INPUT_PATH<br>-out:path:pdb $PDB_OUTPUT_PATH<br>-out:prefix $RELAX_PREFIX<br>-nstruct 1000<br>-relax:thorough

Supplementary Table 5: Rosetta enzyme_design flags and corresponding resfile

enzyme_design flags	resfile
-database $DATABASE_PATH<br>-s $PDB_INPUT_PATH<br>-resfile $RESFILE_INPUT_PATH<br>-out:path:pdb $PDB_OUTPUT_PATH<br>-out:prefix $DESIGN_PREFIX<br>-nstruct 750<br>-favor_native_res 2<br>-linmem_ig 20<br>-enzdes:cst_opt<br>-enzdes:bb_min<br>-enzdes:bb_min_allowed_dev 1.0<br>-enzdes:cst_design<br>-enzdes:cst_min<br>-enzdes:chi_min<br>-enzdes:lig_packer_weight 2.5<br>-enzdes:design_min_cycles 7<br>-packing:ex1<br>-packing:ex2<br>-packing:ex1aro<br>-packing:ex1aro:level 6<br>-packing:extrachi_cutoff 1<br>-packing:linmem_ig 20<br>-packing:soft_rep_design	NATAA<br>START<br>90 A ALLAA<br>91 A ALLAA<br>92 A ALLAA<br>93 A ALLAA<br>94 A ALLAA<br>95 A ALLAA<br>96 A ALLAA<br>97 A ALLAA<br>98 A ALLAA
